# Adsorption of Macrolide Antibiotics by Aged Microplastics of Different Sizes: Mechanisms and Effects

**DOI:** 10.3390/nano15060467

**Published:** 2025-03-19

**Authors:** Qi Li, Jingnan Tan, Haichao Sha, Ke Li, Xi Li

**Affiliations:** Xi’an Key Laboratory of Environmental Simulation and Ecological Health in the Yellow River Basin, College of Urban and Environmental Sciences, Northwest University, Xi’an 710127, China; 202332457@stumail.nwu.edu.cn (J.T.); like1728304402@163.com (K.L.); xili@nwu.edu.cn (X.L.)

**Keywords:** polystyrene, potassium persulfate, azithromycin, clarithromycin, erythromycin

## Abstract

Microplastics (MPs) and antibiotics are widely detected in water bodies. However, the adsorption behavior and mechanism of different particle size polystyrene (PS) MPs on macrolide antibiotics under natural aging remain to be elucidated. In this study, potassium persulfate (K_2_S_2_O_8_) was used to simulate the natural aging process of PS MPs. The adsorption behavior and mechanism of different size PS (80 and 400 μm) toward azithromycin (AZI), clarithromycin (CLA), and erythromycin (ERY) were investigated. Results of SEM showed that the surface roughness of aged PS MPs increased with the appearance of cracks, pits, and pores. XPS and FTIR analyses showed enhanced C=O functional groups in the aging process. The adsorption isotherm models revealed that the aging processes enhanced the AZI, CLA, and ERY adsorption tendency, as evidenced by the highest adsorption capacity for aged-80 μm (645, 665, 184 mg/kg) > original-80 μm (412, 420, 120 mg/kg), and aged-400 μm (280, 330, 110 mg/kg) > original-400 μm (197, 308, 100 mg/kg). Kinetic model fitting revealed that the adsorption process occurred in three stages: rapid, slow, and saturation. Adsorption kinetic curves for original and aged PS MPs conformed to the pseudo-second-order kinetic model. In contrast, the adsorption isotherm data fit the Langmuir model, indicating that the process primarily involved uniform monolayer chemical adsorption. Our findings provide insights into the substantial changes in the interactions between PS and macrolide antibiotics with aging processes.

## 1. Introduction

Since the 1950s, the widespread production and frequent use of plastic products have led to a progressively severe issue of plastic pollution, showing a continuous upward trend. Plastic waste could not decompose completely because it was acid-resistant, alkali-resistant, and contained corrosion-resistant polymer materials. Instead, it gradually broke down into micrometer- or nanoscale plastic fragments through biological and abiotic processes [1]. Microplastics (MPs) were first introduced in *Science* in 2004 [2]. The U.S. Atmospheric Administration defined plastic particles smaller than 5 mm in diameter as MPs [3]. Later, researchers classified plastic particles smaller than 1 µm as nanoplastics [4]. Studies have demonstrated that MPs could interact with various environmental pollutants and could adsorb the COVID-19 [5]. Moreover, MPs could pose potential threats to human health, independently or through interactions with biomolecules.

Antibiotics possess anti-pathogens and other bioactive functions that can interfere with the normal growth and development of cells [6]. Due to their broad-spectrum activity and high efficacy, antibiotics have become the most commonly used class of drugs in treating infections. They inhibit or eliminate pathogenic microorganisms, making them core drugs for preventing and treating infectious diseases. In addition, antibiotics served multiple functions, including treating tumors, immunosuppression, and pest control, which made them indispensable in modern medicine. However, the application of antibiotics carries both advantages and disadvantages. When antibiotics were introduced into the soil, they altered the number and diversity of soil microorganisms and affected the physical and chemical properties of the soil [7]. Wastewater containing azithromycin (AZI), when discharged into streams, led to a significant increase in the number of Firmicutes and Bacteroidetes in the sediment, while simultaneously impacting the microbial community structure [8]. Shen et al. [9] investigated the relationship between quinolone antibiotics in Baiyangdian sediment and the structure and diversity of microbial communities, revealing that antibiotic contamination significantly reduced microbial community abundance and diversity. Therefore, selecting antibiotics correctly and rationally is crucial.

MPs undergo aging and degradation due to various factors, including ultraviolet light, temperature, and wind. The adsorption capacity of MPs for antibiotics is influenced by the characteristics of both the MPs and the antibiotics, making it a complex and multifaceted phenomenon. The adsorption process involves multiple interaction forces determining the binding mode and strength between the two, with hydrophobicity and hydrophilicity being the key binding mechanisms. Hydrophobicity was considered the dominant factor in the interaction between MPs and hydrophobic organic pollutants [10]. Li et al. [11] conducted a thorough study of the adsorption behavior of five antibiotics on the MPs surface. They revealed a positive correlation between the adsorption capacity of the same MPs for these antibiotics and their hydrophobicity. Zhang et al. [12] found that polystyrene (PS) nanoplastics (nano-PS and nano-PS-COOH) contain different functional groups with significantly higher adsorption capacities for the fluoroquinolone antibiotics norfloxacin and levofloxacin. This adsorption mainly occurs through physical means involving polar interactions, electrostatic effects, and hydrogen bonding. Yang et al. [13] discovered that Ca^2+^ and Mg^2+^ interacted with antibiotics on the MPs surface under electrostatic and van der Waals forces, forming a competitive adsorption effect and decreasing the adsorption capacity of MPs for antibiotics. Shen et al. [14] investigated the effects of pH, temperature, and aging on the adsorption capacity of MPs for antibiotics. Their results showed that hydrophobicity increased when organic pollutants adsorbed MPs surfaces, but the adsorption capacity for antibiotics decreased. The adsorption of antibiotics on MPs in the natural environment significantly differed from laboratory simulations. The research on MPs adsorption in the natural environment remained insufficient, with many gaps yet to be filled. Most studies focused on the adsorption characteristics of tetracycline antibiotics, while research on macrolide antibiotics was relatively scarce. PS has been widely used in food packaging and food service, and is regarded as one of the most common types of plastic debris in coastal marine environments [15]. Additionally, previous research has confirmed the superior aging performance of PS compared with polypropylene (PP) and higher adsorption capacity for tetracycline than polyethylene (PE) and PP [16]. Therefore, this study selected PS as the research object to study the adsorption behaviors and adsorption mechanisms of azithromycin (AZI), clarithromycin (CLA), and erythromycin (ERY) by PS under the aging of potassium persulfate (K_2_S_2_O_8_).

## 2. Materials and Methods

### 2.1. Experimental Materials

The PS MPs used in this study had particle sizes of 80 and 400 μm and were purchased from Baitai Biotechnology Co., Ltd. (Beijing, China). The MPs were first soaked in 10% HCl for up to 24 h. Subsequently, they were thoroughly washed with deionized water and placed in an oven at 50 °C for drying. After drying, the MPs were collected for further use [17]. K_2_S_2_O_8_, KBr, NaOH, HCl (36–38%), and algin were purchased from Sinopharm Group Reagent Co., Ltd. (Beijing, China). Absolute ethyl alcohol (HPLC grade) was obtained from Tianjin Tianli Reagent Co., Ltd. (Tianjin, China). NaCl was purchased from Tianjin Ruijinent Reagent Co., Ltd. (Tianjin, China). AZI (HPLC grade), CLA (HPLC grade), and ERY (HPLC grade) were purchased by Shanghai Yuanyuan Biotechnology Co., Ltd. (Shanghai, China). Sodium humate (CP grade) was obtained from Shanghai Aladdin Biochemical Technology Co., Ltd. (Shanghai, China) and utilized for the preparation of humic acid (HA) solution. Unless otherwise stated, all reagents were of analytical grade.

### 2.2. MPs Aging Experiment

K_2_S_2_O_8_ was used to simulate the natural aging process of PS MPs. A total of 2.0 g of 80 and 400 μm PS samples were accurately weighed and placed into flasks, respectively. Subsequently, 80 mL of 0.1 M K_2_S_2_O_8_ solution was added, and the pH of the solution was adjusted to 7 with HCl or NaOH. The solution was continuously stirred at 25 ± 1 °C. Since K_2_S_2_O_8_ was gradually consumed as an oxidant, an equal volume of K_2_S_2_O_8_ solution was supplemented every 12 h.

Additionally, MPs samples were extracted from the suspension by vacuum filtration every two days and washed with ultrapure water. The treated MPs were then placed into a fresh K_2_S_2_O_8_ solution, and the aging process continued. Following these steps, the PS MPs of different particle sizes were continuously treated for 20 days to obtain the aged MPs, which were dried and sealed away from light. A blank control test was conducted in ultrapure water without K_2_S_2_O_8_.

### 2.3. MPs Characterization Method

The samples were fixed on the sample stage using conductive adhesive tape to ensure that they were tightly pressed and evenly distributed. The surface morphology of the MPs was analyzed by scanning electron microscope (SEM) (Oxford Merlin Compact; Oxford, UK). The specific surface area (BET), pore volume, and pore size of MPs were measured by a BET analyzer (Quantachrome Nova 4200e; Boynton Beach, FL, USA). The degassing temperature was 77.35 K, and the degassing time was 2 h. The dispersion stability of the MPs particles at different pH values was evaluated by Zeta potential analyzer (Zetasizer Nano ZS90; Malvern, UK). Crystal structure and lattice parameters of MPs were determined by an X-ray diffraction analyzer (XRD) (Bruker D8; Berlin, Germany); the scanning range was from 5° to 80°, with a scanning speed of 8°/min, a voltage of 40 kV, and a current of 40 mA. The molecular structure of the MPs was analyzed by a Fourier-transform infrared spectrometer (FTIR) (Bruker Equinox 55; Berlin, Germany) within a wavenumber range of 4000 to 400 cm^−1^ [18]. The elemental composition and chemical states were analyzed by an X-ray photoelectron spectrometer (XPS) (Thermo Scientific ESCALAB 250Xi; Waltham, MA, USA).

### 2.4. Antibiotic Adsorption Experiment

Based on the pre-experiment results, the adsorption test was conducted in a 50 mL centrifuge tube with a solid–liquid ratio of 1:2. The test was performed in a constant-temperature shaker at an ambient temperature of 25 °C, with a shaking speed of 180 rpm.

#### 2.4.1. Adsorption Kinetics Experiment

A total of 20 mg of PS MPs and 40 mL of 20 mg/L AZI solution were added to a 50 mL centrifuge tube. The centrifuge tube was placed in the shaker at a temperature of 25 °C at a speed of 180 rpm for 4 days. During the reaction, 3 mL was sampled at the predetermined time point (1, 2, 4, 8, 12, 24, 36, 48, 60, 72, 96 h), filtered by a 0.22 μm filter. Then, the concentration was measured with a UV visible spectrophotometer to calculate the adsorption capacity (Equation (1)). The adsorption kinetics of CLA and ERY were described in the same manner.

(1) $$Q_{t}=\left(C_{0}-C_{t}\right)\times V/m,$$

where $Q_{t}$ (mg kg^−1^) is the adsorption capacity of MPs at a time “t”; $C_{0}$ (mg kg^−1^) is the initial concentration of antibiotics; $C_{t}$ (mg kg^−1^) is the concentration of antibiotics at the time of sampling; V (L) is the volume of the solution; m is the mass of MPs.

#### 2.4.2. Adsorption Isothermal Experiment

A total of 20 mg of PS MPs and a concentration gradient of 10, 15, 20, 30, and 35 mg/L of AZI solution were added to a 50 mL centrifuge tube. The centrifuge tube was placed in the shaker, the temperature was set at 25 °C, the speed was 180 rpm, and the reaction lasted 4 days. The supernatant (3 mL) was filtered through a 0.22 μm filter. The concentration was determined by a UV visible spectrophotometer, the adsorption amount was calculated, and the isothermal adsorption curve was plotted using the adsorption volume data. The adsorption kinetics of CLA and ERY were described in the same manner.

### 2.5. Adsorption Model

The adsorption kinetics data were interpreted using the pseudo-first-order kinetic model (Equation (2)), the pseudo-second-order kinetic model (Equation (3)), and the intraparticle diffusion model (Equation (4)).

(2) $$\ln\left(q_{e}-q_{t}\right)=\ln q_{e}-k_{1}t,$$

(3) $$\frac{t}{q_{t}}=\frac{1}{k_{2}q_{e}^{2}}+\frac{1}{q_{e}}t,$$

(4) $$Q_{t}=k_{p}\times t^{\frac{1}{2}}+C,$$

where $q_{e}$ is the saturation adsorption capacity of MPs; $q_{t}$ is the adsorption capacity of MPs at time “t”; $k_{1}$ is the pseudo-first-order kinetic reaction rate constant; “t” is the adsorption time (h); $k_{2}$ is the pseudo-second-order kinetic reaction rate constant. $Q_{t}$ is the adsorption capacity of MPs at time “t” (mg kg^−1^); C is the constant for thickness, boundary layer; $k_{p}$ is the intraparticle diffusion rate constant, t is the adsorption time (h); C is the intercept, dimensionless.

The adsorption isotherm test data were interpreted using the Langmuir (Equation (5)) and Freundlich model (Equation (6)).

(5) $$Q_{e}=Q_{m}\frac{k_{l}C_{e}}{1+k_{l}C_{e}},$$

(6) $$Q_{e}=k_{F}C_{e}^{1/n},$$

where $Q_{e}$ is the saturated adsorption amount (mg kg^−1^); $Q_{m}$ is the saturated adsorption amount of monolayer (mg kg^−1^); $C_{e}$ is the antibiotic adsorption equilibrium concentration (mg L^−1^); $k_{l}$ is the adsorption equilibrium constant of Langmuir model (mg L^−1^); $k_{F}$ is the adsorption equilibrium constant of Freundlich model.

## 3. Results and Discussion

### 3.1. Changes in Physical Properties of Pristine and Aged PS MPs

**Figure 1:**
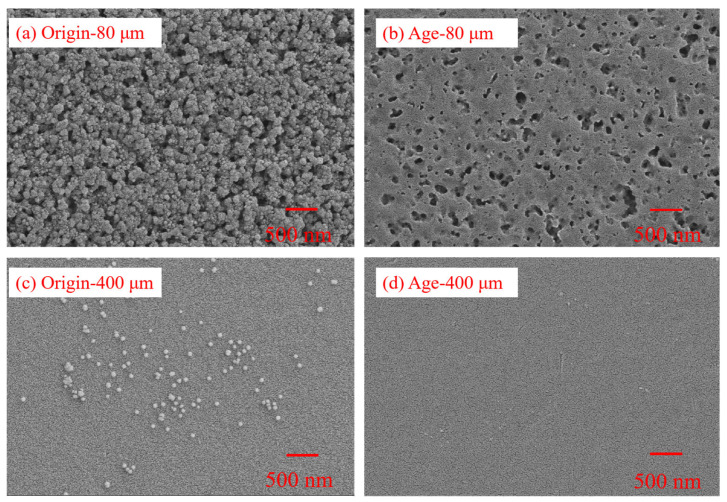
SEM of aging before and after PS MPs with different particle sizes.

The SEM images clearly showed the surface morphology of PS MPs with particle sizes of 80 and 400 μm before and after thermal activation treatment with K_2_S_2_O_8_ in Figure 1. After thermal activation with K_2_S_2_O_8_, the number of surface folds and cracks in both particle sizes increases significantly. The surface of the aging-80 μm PS MPs became rougher, with cracks, pits, and pores observed compared with the original-80 μm PS MPs. Additionally, compared to the original-400 μm PS MPs (106.07 ± 125.69 nm^2^) average cracks area, the internal structure of the aging-400 μm PS MPs (141.51 ± 126.73 nm^2^) was more significant. Xu et al. [19] study found that the surface of aged PS and PE under UV exposure became rough, with the emergence of small holes, cracks, and an increased number of microporous structures. This indicated that the aging process of MPs mainly began with the formation of small pits, pores, and cracks on the surface of the MPs. Moreover, the smaller the particle size, the more pronounced the aging effect.

After aging, the BET of PS with a particle size of 80 and 400 μm increased from 2.6186 and 0.1465 to 6.0697 and 0.6081 m^2^/g. The BET of aging-80 and 400 μm PS increased by approximately 2.33- and 4.15-fold compared with the original-80 and 400 μm PS. A larger BET implied increased active surface sites, which could interact with antibiotic molecules, thereby enhancing the adsorption efficiency [20]. This suggested that aging increased the BET of MPs.

For MPs, the positive or negative value of the Zeta potential not only revealed the characteristics of surface groups and charge distribution, but it also provided important insights into the interactions between MPs and other substances in the environment. The isoelectric points of the original- and aged-80 μm PS MPs were 5.75 and 6.73, respectively, while the isoelectric points of the original- and aged-400 μm PS MPs were 4.82 and 6.32, respectively. Notably, the Zeta negative potential of the aged PS MPs significantly increased compared to the original-PS, indicating a substantial enhancement of the negative charge on the surface due to the aging process [21]. This suggested that the aging process also caused changes in the surface charge of MPs.

**Figure 2:**
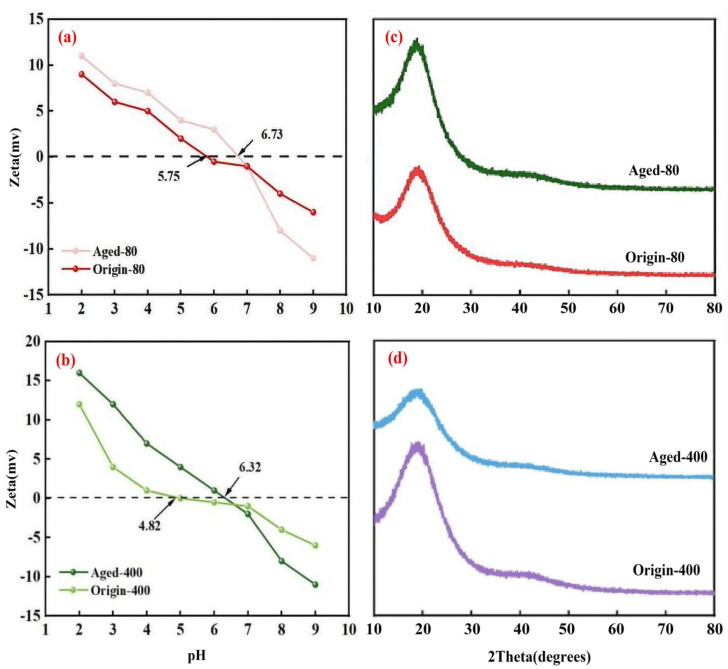
Zeta (**a**,**b**) and XRD (**c**,**d**) of aging before and after PS MPs with different particle sizes.

XRD of PS MPs before and after aging was shown in Figure 2. PS had sharp diffraction peaks and weak sharp fronts at high angles, indicating that PS had high crystallinity. Compared with the original PS, the position of the diffraction peak of the aged PS did not shift significantly, which meant that the aging process did not change the basic type of crystal structure of PS. However, the intensity of the diffraction peak increased significantly after aging, which might be due to the aging process increasing the order and the crystallinity of the crystal structure of PS MPs [22]. Therefore, it could be inferred that the aging process enhanced the crystallinity of PS.

### 3.2. Changes in Chemical Properties of Pristine and Aged PS MPs

The FTIR spectrum of PS MPs with different particle sizes before and after aging was shown in Figure 2. The original-80 μm PS showed absorption peaks at 3444 and 2920 cm^−1^, corresponding to the –OH stretching vibration [23] and the asymmetric and symmetric stretching vibrations of the –CH_2_ group, respectively. Compared to the original-80 μm PS, the aged-80 μm PS showed an increase in the intensity of the absorption peak at 1438 cm^−1^, indicating stronger C–H stretching vibrations [24]. An enhancement of the absorption peak at 1698 cm^−1^ was also observed, which was attributed to the stretching vibration of the carbonyl group, suggesting the formation of carbonyl groups during aging due to chain scission and free radical oxidation [25]. The original-400 μm PS and aged-400 μm PS showed absorption peaks at 2935 cm^−1^, corresponding to the –CH_2_ group. Absorption peaks related to the external and internal deformations of the benzene ring C–H were observed in the 800–900 cm^−1^ region. Absorption peaks related to the bending vibration of the benzene ring (C=C) were found at 1456, 1617, and 1696 cm^−1^. However, the absorption peak intensity of the aged-400 μm PS was significantly higher.

Furthermore, due to the external and in-plane deformations of the benzene ring, the related absorption peaks of PS with both particle sizes changed after aging, with the intensities significantly increasing [26]. Wang et al. [27] found that after UV exposure, the surface functional groups (–OH and C=O) of PE and PS were significantly strengthened. The aging process increased the number of C–H, –OH, and other functional groups on the MPs surfaces, enhancing their hydrophilicity and increasing their likelihood of interacting with water molecules. In addition, Belmont et al. [28] suggested that UV aging could enhance the hydrophilicity of MPs, thereby affecting their adsorption performance for antibiotics. The infrared spectroscopy results indicated that the surface functional groups of MPs changed during aging, which may have impacted their hydrophilicity and, consequently, their adsorption capacity for antibiotics.

**Figure 3:**
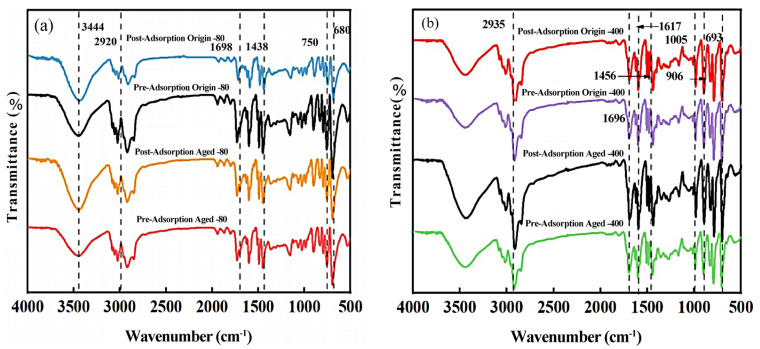

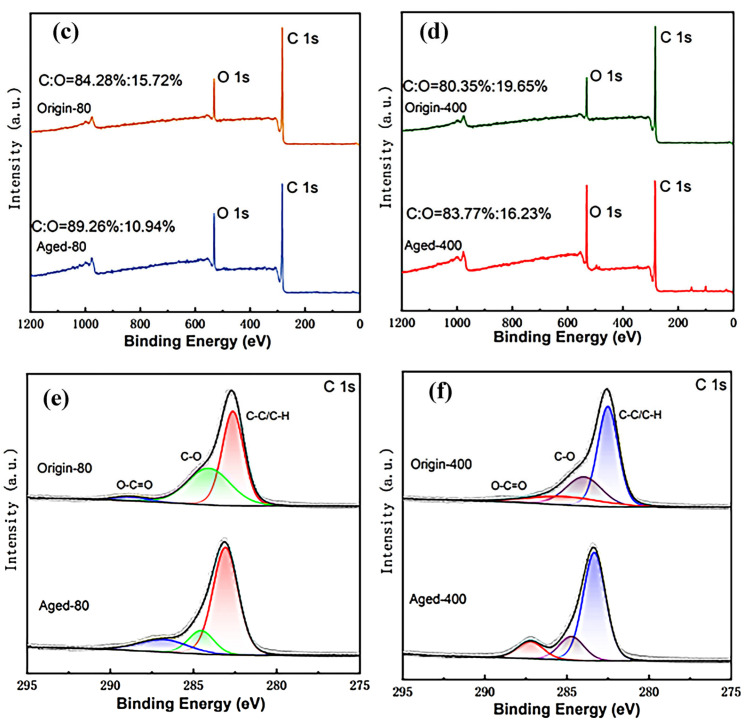
FTIR diagram before and after aging and adsorption AZI (**a**,**b**) of PS MPs with different particle sizes. XPS (**c**–**f**) diagram before, after aging of PS MPs with different particle sizes.

Gaining further insight into the changes in the elemental composition of PS MPs surfaces through XPS analysis was crucial for understanding the modifications in their chemical properties. Figure 3 showed the XPS results of PS MPs with different particle sizes before and after aging. In the full spectrum, both the original- and aged-PS MPs exhibited peaks corresponding to C 1s (278 eV) and O 1s (528 eV), indicating the presence of C and O elements, consistent with their chemical structure. The O/C ratio was an indicator used to evaluate the degree of aging in MPs [29]. The O/C ratio of the aged-80 and 400 μm PS MPs increased by 4.96% and 3.42% compared to the aged-80 and 400 μm PS MPs. The increase in the O/C ratio indicated that more oxygen-containing functional groups were formed on the surface of the MPs during the oxidation process. The O element mainly showed two characteristic peaks in the XPS full spectrum: C–O and O–C=O. For the two different particle sizes of PS MPs, their C 1s spectra showed similar trends after aging. The peak area of O–C=O significantly increased, while the peak area of C–O decreased. These two changes indicated an increase in the number of essential functional groups on the MPs surface and a reduction in alkyl functional groups on the MPs surface. The decrease in alkyl groups might have been related to their consumption during the oxidation process and the subsequent formation of new oxygen-containing functional groups. This suggested that the aging process led to a transformation in the surface chemical structure.

### 3.3. Adsorption Behavior

#### 3.3.1. Adsorption Kinetics

**Figure 4:**
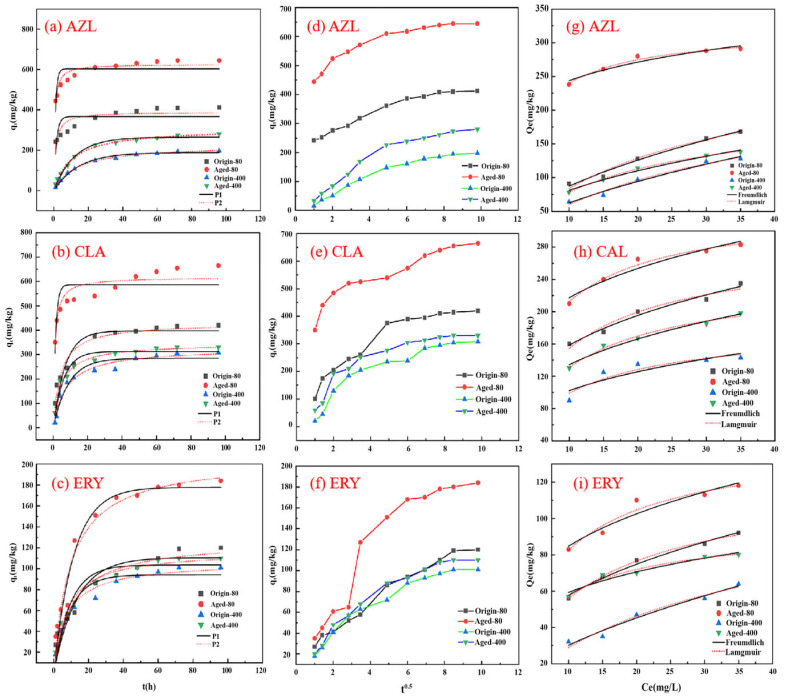
Adsorption kinetics and isotherm models of AZI, CLA, and ERY onto original-PS and aged-PS: (**a**–**c**) kinetics; (**d**–**f**) Weber–Morris; (**g**–**i**) isotherm models.

The adsorption kinetic curves of PS MPs with two particle sizes on macrolide antibiotics were shown in Figure 4. The AZI, CLA, and ERY adsorption processes on PS MPs can be divided into three stages. In the first 24 h, the process followed a rapid adsorption phase. During this period, the adsorption rate quickly reached its maximum due to the initial concentration gradient and mass transfer driving force [30]. From 24 to 48 h, the adsorption rate slowed, primarily because the diffusion rate within the particles decreased, leading to a gradual reduction in the overall adsorption rate. Finally, adsorption reaches equilibrium at approximately 96 h. The adsorption capacities of the PS MPs to AZI, CLA, and ERY were as follows: aged-80 μm (645, 665, 184 mg/kg) > original-80 μm (412, 420, 120 mg/kg) and aged-400 μm (280, 330, 110 mg/kg) > original-400 μm (197, 308, 100 mg/kg). These results indicated that both 80 and 400 μm aged-PS MPs showed enhanced AZI, CLA, and ERY adsorption compared to original-PS MPs, especially the aged-80 μm PS MPs. The results also showed that the adsorption capacity of PS MPs with a particle size of 80 μm was significantly higher than that of 400 μm PS. The smaller size of the MPs provided more potential adsorption sites, leading to stronger interactions with AZI, CLA, and ERY. Therefore, PS MPs with smaller particle sizes exhibited an increased capacity to adsorb AZI, CLA, and ERY.

To better understand the adsorption process of macrolide antibiotics, the experimental results were analyzed using the quasi-first-order and quasi-second-order kinetic models, with the corresponding parameters shown in Table 1. The analysis indicated that the quasi-second-order kinetic model provided better fit AZI, CLA, and ERY adsorption on the PS MPs surface. This suggests that multiple factors influenced the adsorption process, and there are complex interactions between the adsorbent and adsorbate, including nonspecific van der Waals forces and π–π bond interactions. After reaching adsorption equilibrium, the aged PS MPs showed an increased adsorption capacity for the AZI, CLA, and ERY antibiotics compared to the original-PS MPs of the same particle size. The adsorption capacities of aged-80 μm PS for AZI, CLA, and ERY increased from 389.66, 429.57, and 127.01 mg/kg to 629.19, 616.78, and 205.49 mg/kg compared to original-PS MPs. For 400 μm PS, the adsorption capacities for AZI, CLA, and ERY increased from 225.03, 326.43, and 106.58 mg/kg to 309.48, 344.79, and 117.11 mg/kg. This confirmed that PS MPs enhanced their pollutant adsorption capacity during aging.

The intraparticle diffusion models were presented in Figure 4. The adsorption process of AZI, CLA, and ERY by original- and aged-PS MPs could be divided into three stages. In the first stage, the MPs rapidly attracted antibiotic molecules due to the large concentration gradient between the antibiotic and the MPs phases. In the second stage, the adsorption rate slowed down significantly. As the adsorption process reached the final stage, it gradually approached saturation. From Table 1, the diffusion coefficient (k) of the aged MPs is higher than that of the original MPs. This was likely due to the decreased particle size and increased availability of adsorption sites on the surface of the aged MPs. The inner pores of the MPs particles were shorter, facilitating the faster diffusion of antibiotic molecules to the adsorption sites, further accelerating the adsorption process [31].

#### 3.3.2. Adsorption Isotherm Behavior

The Freundlich and Langmuir models were presented in Figure 4 and Table 1. The adsorption of antibiotics by MPs increased proportionally with the increasing concentration of antibiotics in the solution, exhibiting a significant nonlinear relationship. Aged-PS MPs demonstrated more substantial adsorption capacity than the original-PS under different initial concentration conditions. Additionally, the adsorption rate of these three macrolide antibiotics on PS followed a pattern of initial increase with increasing antibiotic concentration, forming a typical “S” shaped curve. The adsorption amounts of the three macrolide antibiotics on PS were ranked as follows: aged-80 μm > original-80 μm > aged-400 μm > original-400 μm.

Based on the data in Table 1, both 80 and 400 μm PS MPs exhibited better adsorption of AZI according to the Langmuir model. This suggested that multilayer adsorption is not significant and homogeneous monolayer physical adsorption predominates. This result may have been attributed to the experimental pH of 7, at which AZI exists in a zwitterionic form. The negative charge on the surface of the PS MPs interacts with the positively charged groups on the antibiotic surface, reducing the likelihood of multilayer adsorption. It was also observed that smaller MPs particle sizes resulted in a higher adsorption equilibrium constant (K_F_). A higher K_F_ value indicates a stronger affinity during the adsorption process.

Additionally, the K_F_ values for aged-PS MPs were significantly higher than those for their original-PS MPs, suggesting that the aging process with K_2_S_2_O_8_ enhanced the affinity of PS MPs for antibiotics [32]. As shown in Table 1, the n values for the Freundlich model fitting were all below 1, indicating that the adsorption capacity of PS MPs decreased as the initial antibiotic concentration increased. Moreover, the n value for 400 μm PS was more significant than that for 80 μm PS, suggesting that stronger adsorption corresponds to a lower n value.

The adsorption process followed a nonlinear isothermal model, indicating that hydrophobic interactions do not solely govern the adsorption mechanism. Other factors, such as electrostatic interactions and hydrogen bonding, might also have played a significant role in the process [27]. Fan Xiulei et al. [33] found that the Freundlich model better described the adsorption process of Tire wear particles on Oxytetracycline and Sulfamethoxazole before and after aging. Additionally, Yang et al. [13] studied the adsorption of Tetracycline on PE, PS, and polyamide MPs, and found that the adsorption process and the Langmuir isothermal model fitted the experimental results better. Furthermore, changes in adsorption behavior may also have been influenced by factors such as solution pH and ionic strength. These factors could affect the ionic form of antibiotics, the surface properties of PS MPs, and the interactions between them.

### 3.4. The Effects of Environmental Factors

#### 3.4.1. Ionic Strength

Ionic strength was a key factor influencing the adsorption performance of MPs. Kinetic and isotherm adsorption experimental data showed that 80 μm PS MPs had a more substantial adsorption capacity than 400 μm PS MPs. Therefore, 80 μm PS MPs were selected for subsequent experiments. Within the concentration range of 0.5‰, 10‰, 20‰, and 35‰, the adsorption of AZI by PS MPs increased with the NaCl concentration, initially rising and then leveling off. When the NaCl concentration exceeded 10‰, the adsorption capacity no longer increased, indicating that high concentrations of Na^+^ competed with antibiotic molecules for adsorption sites on the PS surface.

**Figure 5:**
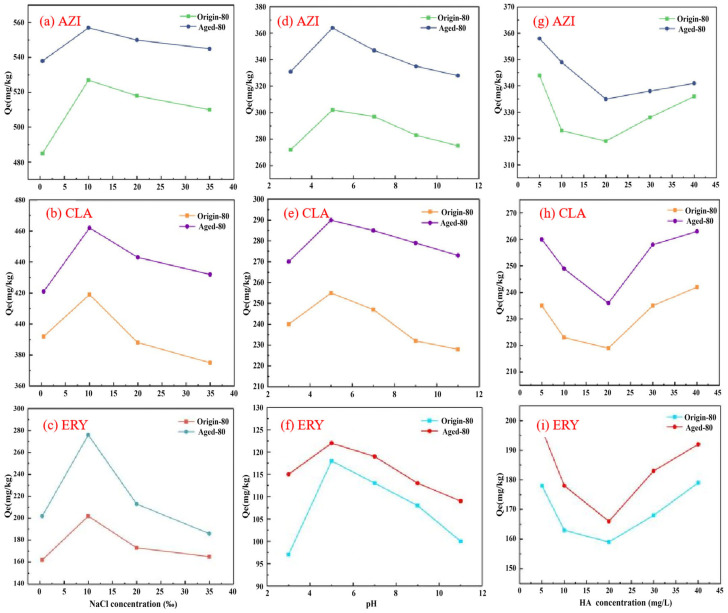
The effect of ionic strength, pH, and HA on AZI, CLA, and ERY adsorption onto original- and aged-PS (80 μm): (**a**–**c**) NaCl; (**d**–**f**) pH; (**g**–**i**) HA.

Additionally, the high ion concentration in the NaCl solution formed a double-layer structure through electrostatic effects. This increased the thickness of the double layer and weakened the electrostatic interactions, hindering the diffusion of antibiotic molecules to the MPs surface. As a result, the adsorption amount decreased. For CLA and ERY, the adsorption by PS MPs initially increased and then decreased as the NaCl concentration increased. As shown in Figure 5, at a NaCl concentration of 10‰, the salting-out effect reduced the solubility of the antibiotics in the solution, enhancing their hydrophobicity. This increased the hydrophobic interaction between the PS MPs and the antibiotics, promoting adsorption.

However, as the Na^+^ concentration increased, competitive adsorption became more prominent, resulting in a decrease in antibiotic adsorption. Na^+^ could have replaced H^+^ in the acidic functional groups on the surface of the MPs, hindering hydrogen bond formation and further reducing the adsorption capacity of PS MPs for the antibiotics. Costigan et al. [34] comprehensively reviewed the adsorption of organic pollutants by MPs. They suggested that hydrophobicity may play a more significant role in solutions with higher salt content, while at low hydrophobicity, adsorption was more significant in freshwater systems. When the NaCl concentration reached higher levels, the viscosity of the solution increased significantly. The ionic interactions strengthened, and the fluidity of the solution decreased. This hindered the diffusion of pollutants and their contact with the MP surface. As the Na^+^ concentration increased, the adsorption sites on the PS MP surface gradually became saturated. At a NaCl concentration of 10‰, the competitive adsorption between antibiotic molecules and Na^+^ reached equilibrium. The adsorption sites on the MP surface were jointly occupied by antibiotics and Na^+^, resulting in a relatively stable amount of adsorption. However, Xu et al. [35] found that when salinity increased from 0.05% to 3.5%, the adsorption of tetracycline by MPs did not change significantly. Gigault et al. [36] discovered that nano-PS exhibited stability in a 350 mM NaCl solution. When the NaCl concentration exceeded 500 mM, irreversible aggregation occurred, causing the nanoparticles to become more compact and unfavorable for adsorption. This indicated that the presence of Na^+^ affected the adsorption efficiency of PS MPs for antibiotics.

#### 3.4.2. pH

The pH of the solution was an important environmental factor that affected the surface charge of MPs and the ionization state of pollutants. Figure 5 showed that as the pH increased, AZI, CLA, and ERY adsorption onto PS MPs first increased and then decreased, with the maximum adsorption occurring at pH 5. Mejías et al. [37] investigated the adsorption of four macrolide antibiotics and one metabolite onto two types of MPs. They found that pH affected the adsorption, with the maximum adsorption occurring at a neutral pH, which was consistent with our experimental results. Both the tested pH range, PS, and aged-PS surfaces exhibited inherent negative charges within the tested pH range, which became more pronounced as the pH increased. This indicated that changes in pH altered the charge characteristics of the MPs surfaces. On the other hand, pH changes influenced the ionic forms or solubility of the adsorbates. Under the experimental conditions, when the pH reached 3, the adsorption of AZI onto positively charged MPs decreased due to electrostatic repulsion. When the pH reached 5, the adsorption capacity increased due to electrostatic attraction between AZI and negatively charged MPs. Once the pH exceeded 7.3, AZI existed in its anionic form and experienced electrostatic repulsion with the MPs surface, which decreased its adsorption capacity. Under acidic conditions, the positive charge on the MPs surface repelled the cationic AZI. Additionally, H^+^ ions in the strongly acidic environment competed with the cations for adsorption sites. However, as the pH increased, this competitive effect weakened, thus increasing the adsorption capacity. In conclusion, electrostatic interactions played distinct roles in adsorption under different pH conditions.

#### 3.4.3. HA

HA was a water-soluble organic substance rich in functional groups such as carboxyl and hydroxyl groups. It could form complexes with heavy metals and organic pollutants, thereby affecting the migration and degradation of these pollutants in the environment [38]. When both MPs and HA were introduced into antibiotic solutions, the effect of HA concentration on the adsorption of macrolide antibiotics onto PS MPs was analyzed. The results, shown in Figure 5, demonstrated that under low concentration conditions, the adsorption of the three macrolide antibiotics onto PS MPs gradually decreased as the HA concentration increased. As a large organic molecule, HA had a strong adsorption capacity and competed with antibiotics for adsorption sites, thus inhibiting antibiotic adsorption. At low concentrations, the competitive effect of HA was more significant. The adsorption of AZI by the original-80 μm PS and aged-80 μm PS MPs decreased from 698 mg/kg and 844 mg/kg to 562 mg/kg and 787 mg/kg, respectively. The adsorption of CLA decreased from 435 mg/kg and 520 mg/kg to 360 mg/kg and 410 mg/kg, respectively. The adsorption of ERY decreased from 446 mg/kg and 485 mg/kg to 387 mg/kg and 409 mg/kg, respectively. When the HA concentration exceeded 20 mg/L, the adsorption capacity of the antibiotics on the MP surface gradually increased. This was because humic substances formed a coating on the MP surface. The oxygen-containing functional groups in the humic substances interacted with the antibiotics through cation-π bonding and π–π donor-acceptor interactions, promoting adsorption.

### 3.5. Adsorption Mechanism

The SEM images revealed that the aged PS exhibited more wrinkles and cracks, with a rougher surface accompanied by pits and pores. This unique structure provided more adsorption sites, which enhanced its adsorption capacity compared to the original PS. Therefore, the pore structure largely influenced the adsorption performance of the MPs. Furthermore, compared to the original MPs, the BET of the aged 80 μm and 400 μm PS MPs increased by 2.33 times and 4.15 times, respectively. Additionally, as the particle size of the MPs decreased, their BET, pore volume, and pore size increased. The increase in BET led to a higher number of active sites on the surface of the MPs, which interacted with antibiotic molecules, thus improving the adsorption efficiency [39].

The FTIR spectra of PS MPs did not show significant changes before and after the adsorption of AZI (Figure 3a,b). Therefore, physical adsorption was likely the primary adsorption mechanism. After aging, the negative charge on the surface of the MP increased, and the smaller the particle size, the greater the electronegativity. This further indicated that electrostatic interactions were one of the factors influencing the adsorption of positively charged antibiotics by aged MPs [40]. Aging also enhanced the crystallinity of PS. Consequently, the adsorption behavior of the MPs toward pollutants might have been influenced by changes in crystallinity. During the aging process, the oxygen-containing functional groups on the surface of the MPs changed, and the role of hydrogen bonding in the adsorption process might have been strengthened. Electrostatic interactions played a key role in the adsorption of antibiotics by the MPs. Adsorption kinetic experiments also confirmed that the MPs exhibited better adsorption performance toward antibiotics after aging. Smaller particles exhibited better adsorption efficiency than larger ones. The maximum adsorption capacity for AZI and CLA was significantly higher than that for ERY. This might have been due to more functional groups, such as carboxyl and hydroxyl groups, in AZI and CLA compared to ERY. These functional groups enhanced the binding ability between the MPs and antibiotics, making it easier for these two antibiotics to be rapidly adsorbed by the coexisting MPs.

The pseudo-second-order kinetic model better suited AZI, CLA, and ERY adsorption onto PS MPs. This indicated that various factors influenced the adsorption and that the adsorption relationship between the adsorbent and the adsorbate was complex, involving non-specific van der Waals forces and π–π interactions. Compared to the original MPs, the KPI values of the aged MPs were higher, likely due to the decrease in particle size and the increase in adsorption sites during the aging process. The shorter pore channels inside the MP particles facilitated the faster arrival of antibiotic molecules at the adsorption sites, further accelerating the adsorption process. The Langmuir model better described the adsorption of AZI onto both 80 and 400 μm PS MPs. This indicated that multilayer adsorption was insignificant under these conditions, and that monolayer homogeneous physical adsorption dominated. The negative charge on the surface of the PS MPs interacted with the positive functional groups on the antibiotic surface, which reduced the possibility of multilayer adsorption. The adsorption process followed a nonlinear isotherm model, indicating that hydrophobic interactions did not solely drive the adsorption but were also influenced by electrostatic interactions and hydrogen bonding effects. In addition, the adsorption behavior of PS under the influence of ionic strength, pH, and HA conditions suggested that the adsorption mechanism between PS, aged-PS, and macrolide antibiotics was mainly driven by electrostatic forces and micropore filling, which were characteristic of physical adsorption.

## 4. Conclusions

This study investigated the adsorption behavior and characteristics of PS MPs and three typical macrolide antibiotics under aging conditions induced by thermal activation of K_2_S_2_O_8_. Two sizes of PS MPs, 80 μm and 400 μm, were selected, and three macrolide antibiotics, AZI, CLA, and ERY, were chosen as target pollutants for batch adsorption equilibrium experiments. SEM images of the aged PS MPs, treated with thermal K_2_S_2_O_8_, revealed a rougher surface with increased folding, cracks, pits, and pores. The surface morphology appeared more fragmented, with smaller MP particles exhibiting a more pronounced roughness. XPS and FTIR analysis of the MPs before and after aging showed an increase in the intensity of the C=O functional group peaks and an upward trend in the O/C ratio. These changes suggested the formation of more oxygen-containing functional groups during the oxidation process, which enhances the hydrophilicity of the MPs and facilitates antibiotic adsorption. The aging process significantly improved the adsorption capacity of PS MPs for macrolide antibiotics. This study demonstrated that aging increases the adsorption capacity of PS MPs, with smaller particles (80 μm) showing a higher adsorption capacity than larger ones (400 μm) for all three antibiotics. An adsorption kinetics fitting revealed that the adsorption process could be divided into three stages: an initial rapid adsorption stage, a slower adsorption stage, and finally, the adsorption saturation stage. The quasi-second-order kinetic model best fitted the adsorption process for the original and aged PS MPs, indicating that chemical adsorption and various factors influenced the process. The adsorption isotherms followed the Langmuir model, suggesting that the adsorption of the three antibiotics on PS MPs was dominated by uniform monolayer chemisorption. Electrostatic interactions and competitive adsorption were found to be key factors affecting the adsorption capacity of the MPs. This study comprehensively investigated the adsorption behaviors and mechanisms of PS MPs with different particle sizes toward three macrolide antibiotics under K_2_S_2_O_8_ aging. Future studies should consider incorporating a broader range of MPs types to further prove the adsorption behaviors and mechanisms between MPs and macrolide antibiotics under K_2_S_2_O_8_ aging.

## Figures and Tables

**Table 1:** Parameters calculated with three different kinetic models and isotherm models for the adsorption of AZI, CLA, and ERY onto original-PS and aged-PS.

Models	Antibiotic	AZI				CLA				ERY			
	PS-MPs	Original-80	Aged-80	Original-400	Aged-400	Original-80	Aged-80	Original-400	Aged-400	Original-80	Aged-80	Original-400	Aged-400
Pseudo-first-order	K_1_ (min^−1^)	0.675	1.046	0.069	0.083	0.144	0.734	0.112	0.166	0.076	0.087	0.108	0.107
	q_e1_ (mg/kg)	366.87	602.9	188.81	264.78	397.34	586.28	284.39	312.58	110.59	177.88	94.204	103.643
	R^2^	0.481	0.592	0.988	0.986	0.884	0.663	0.952	0.954	0.881	0.956	0.937	0.946
Pseudo-second-order	K_2_ × 10^−4^	0.0024	0.0029	3.444	3.105	5.488	0.0018	4.001	6.352	7.82	5.09	0.001	0.001
	q_e2_ (mg/kg)	389.66	626.19	225.03	309.48	429.57	616.78	326.43	344.79	127.01	205.49	106.58	117.11
	R^2^	0.93	0.973	0.998	0.995	0.971	0.966	0.993	0.992	0.97	0.972	0.989	0.983
Weber–Morris	K_1_ (min^−1^)	28.5	58.47	38.05	49.3	73.02	86.37	11.4	88.94	12.584	16.69	21.09	21.67
	C_1_	213.64	390.86	/	/	49.03	292.35	/	/	16.71	21.26	/	0.052
	R_1_^2^	0.973	0.946	0.989	0.996	0.892	0.867	0.971	0.878	0.942	0.895	0.996	0.948
	K_2_ (min^−1^)	22.22	23.73	20.2	23.156	26.98	28.98	18.53	20.79	19.68	84.87	30.1	38.47
	C_2_	246.21	518.42	41.199	97.24	150.7	421.35	130.28	187.83	12.22	12.98	9.18	9.2
	R_2_^2^	0.977	0.966	0.984	0.95	0.802	0.908	0.881	0.977	0.934	0.945	0.967	0.954
	K_3_ (min^−1^)	1.89	2.15	5.43	8.8	4.74	11.65	2.15	5.86	2.93	4.39	0.86	1.72
	C_3_	393.51	624.64	144.85	194.95	373.78	552.22	251.78	309.64	78.19	155.17	84.717	101.85
	R_3_^2^	0.974	0.601	0.817	0.906	0.974	0.926	0.801	0.601	0.688	0.999	0.601	0.601
Langmuir	Q_m_ (mg/kg)	278.7	322.06	244.75	202.32	281.71	328.74	182.08	241.71	121.56	142.82	116.01	95.027
	K_l_ (L/mg)	0.042	0.289	0.032	0.062	0.121	0.182	0.119	0.117	0.081	0.136	0.033	0.155
	R^2^	0.973	0.981	0.978	0.998	0.955	0.981	0.9884	0.983	0.996	0.945	0.96	0.967
Freundlich	K_F_ (g/(mg·min))	25.68	170.73	15.81	29.15	79.049	130.11	51.541	66.402	24.35	45.01	7.756	33.23
	n	0.532	0.154	0.598	0.444	0.302	0.223	0.298	0.307	0.376	0.275	0.592	0.252
	R^2^	0.983	0.931	0.979	0.985	0.975	0.939	0.805	0.976	0.988	0.92	0.972	0.944

## Data Availability

Data are contained within the article.
